# Compassionate Use of Remdesivir in Pregnancy: A Case Series From a COVID-19 Dedicated Center and Review of Literature

**DOI:** 10.7759/cureus.43671

**Published:** 2023-08-17

**Authors:** Priyanka Sharma, Priyanka Gupta, Rashmi Virmani, Anil Pandey, Jagadish C Sharma, Avir Sarkar

**Affiliations:** 1 Obstetrics and Gynecology, Employees' State Insurance Corporation (ESIC) Medical College and Hospital, Faridabad, Faridabad, IND; 2 Pediatrics, Employees' State Insurance Corporation (ESIC) Medical College and Hospital, Faridabad, Faridabad, IND; 3 Anesthesiology, Employees' State Insurance Corporation (ESIC) Medical College and Hospital, Faridabad, Faridabad, IND; 4 Physiology and Molecular Biology, Employees' State Insurance Corporation (ESIC) Medical College and Hospital, Faridabad, Faridabad, IND

**Keywords:** sars-cov-2, prone positioning, tocilizumab, covid-19, pregnancy, remdesivir

## Abstract

Pregnancy is associated with immunological changes that could render an individual vulnerable to the severe coronavirus disease 2019 (COVID-19) disease. Even as we witnessed the third and most widespread pandemic wave, a conclusively advantageous treatment option still remained elusive. Remdesivir was one of the front-running therapeutic options that received emergency use authorization (EUA) and subsequent approval for the management of moderate to severe COVID-19 infections. Here, we report a series of moderate to severe COVID-19-infected pregnancies and the experience of remdesivir use on a compassionate basis. Four cases of pregnancy complicated with moderate to severe COVID-19 infections where remdesivir was administered were recruited into the study, and their outcome was assessed objectively. Of these cases, three women received remdesivir in addition to standard SARS-CoV-2 treatment in the antenatal period. One woman received remdesivir after delivery. One woman received tocilizumab in addition to remdesivir and standard SARS-CoV-2 care. Two women survived and were subsequently discharged to home care. Two succumbed to the disease. One baby who was exposed to remdesivir in utero is doing well at six months post-delivery. Remdesivir had been granted EUA for the treatment of suspected or laboratory-confirmed COVID-19 infection in adults and children who were hospitalized with severe disease or requiring supplemental oxygen and mechanical ventilation or extracorporeal membrane oxygenation (ECMO) in May 2020. This issuance allowed the use of the same dosing regimen in pregnant and parturient women as in the general adult population. Thus, this series of cases tried to assess the outcome of this drug among COVID-19-infected pregnant women. Early initiation of remdesivir in pregnancy in the viremic phase seems to provide some advantages in the survival outcome. Its use may be associated with transient elevation in hepatic transaminases in some cases. No detrimental effects on the ongoing pregnancies, fetuses, or neonates have been observed. Further large-scale studies may provide more conclusive evidence.

## Introduction

The coronavirus disease 2019 (COVID-19) pandemic with its ebbs and flows has wreaked havoc globally. While initial reports indicated the possibility of a milder disease in pregnancy and puerperium, later trends show that the infection can be extremely virulent in antenatal women [1]. Pregnancy-associated immunological changes could be the potential cause, rendering pregnant individuals vulnerable to the severe COVID-19 disease [2-4]. Remdesivir is one of the front-running therapeutic options that received emergency use authorization (EUA) and subsequent approval for the management of moderate to severe COVID-19 pneumonia [5,6]. Through this case series, we tried to highlight the treatment outcomes of adding remdesivir on a compassionate therapeutic use basis for pregnant women admitted with moderate to severe COVID-19 infections.

## Case presentation

Case 1

**Figure 1:**
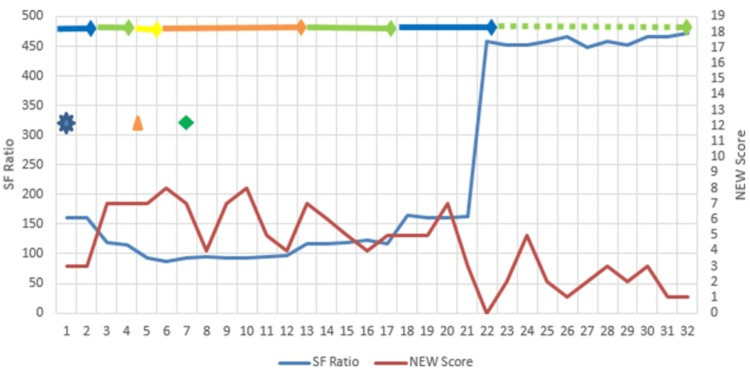
Clinical timeline of case 1. SF ratio = SpO_2_/FiO_2_;_ _NEW Score = National Early Warning Score

A 24-year-old G2P1L1 presented at 37 weeks with shortness of breath for one day. Saturation (SpO_2_) was 96% on room air. Vitals were stable. She tested positive on reverse transcriptase polymerase chain reaction (RTPCR) for SARS-CoV-2 on the same day and was admitted to intensive care unit (ICU) care. Non-stress test (NST) showed non-reassuring fetal heart pattern, and an emergency caesarean section was performed under spinal anesthesia with the birth of a healthy born male weighing 2.8 kg with an Apgar score of 8 and 9 at one and five minutes after birth, respectively (Figure 1). There were two tight loops of cord around the fetal neck. Post operatively, she received supportive COVID-19 care and broad-spectrum antibiotics as per hospital policy. The Key for Clinical Intervention and Outcome is presented in Appendix.

SpO_2_ was 96% on nasal mask. On day two, the patient showed intermittent decline in SpO_2_ and was stepped up to oxygen supplementation with a non-rebreathing mask (NRM), after which SpO_2_ remained above 90%. On day five, she developed acute breathlessness. SpO_2_ declined to 84%, and she was stepped up to oxygen support by high-flow nasal cannula (HFNC). Due to lack of improvement, injection remdesivir was started as a 200 mg loading dose, followed by 100 mg for the next four days after proper counseling and informed consent. Intravenous methylprednisolone was initiated to curb inflammatory response. Prone positioning and low-molecular-weight heparin (LMWH) were also initiated, and respiratory support stepped up to non-invasive ventilation (NIV). Single-dose tocilizumab 400 mg was given on day seven for markedly elevated interleukin 6 (IL-6) levels. The computed tomography (CT) chest score was 12/25.

The patient showed improvement over the following two days. However, on day 10, she had an episode of generalized seizures, which responded to levetiracetam. There was no neurological deficit, and the seizures were provisionally attributed to hypoxemia. Gradual clinical improvement in SpO_2_ and decline in oxygen requirement were seen through day 13, and the patient was shifted to NRM. By day 17, the patient was comfortable with supplemental oxygen by simple nasal cannula and was shifted to the ward. LMWH was stopped, and she transitioned to oral apixaban. She tested RTPCR-negative on day 24. Oral steroids were gradually tapered off. She showed steady improvement, was tapered off intravenous steroids and put on oral apixaban discharged in a satisfactory condition on day 32 of hospital stay. Her neonate was negative for SARS-CoV-2 after birth and was discharged to routine COVID-19 care after birth. The baby is doing well at the 18-month follow-up (Table 1). 

**Table 1:** Patient characteristics, delivery, and neonatal outcome. GA: gestational age; LSCS: lower-segment cesarean section; RTPCR: reverse transcriptase polymerase chain reaction; NICU: neonatal intensive care unit

Case no.	Parity age (years) GA (weeks)	Comorbidity	Duration of symptoms before remdesivir use	Duration of remdesivir use	Adverse events after remdesivir use	Patient survival outcome	Mode of delivery	Liquor	Interval between the loading dose and delivery	APGAR score (1, 5 min)	Need for resuscitation	Baby RTPCR	Baby survival outcome
										Birth weight (kg)	NICU stay		
Case 1	G2P1L1 24 years 38 weeks	Nil	5 days	5 days	None	Alive	LSCS for fetal distress during hospital stay	Clear	Remdesivir started after delivery	8, 9	No	Negative at birth and day two of life	Alive and healthy. Doing well at 6 months age
										2.8 kg	None		
Case 2	G2P1L1 28 years 23 weeks	Nil	2 days	5 days	Possible transient transaminitis	Alive	Undelivered at discharge. Delivered at a private nursing home at 37 2/7 weeks by LSCS for oligohydramnios and intrauterine growth restriction	Clear	14 weeks	9, 10	No	-	Alive and healthy. Doing well at 6 months age
										2.3 kg	None		
Case 3	G3P2L2 35 years 29 weeks	Moderate Anemia	6 days	5 days	None	Death at day 7	Undelivered at time of death	-	-	-	-	-	-
Case 4	G6P2L2A3 27 years 34 weeks	Nil	3 days	5 days	None	Death at day 6	Vaginal delivery during hospital stay	Clear	2 days	9, 10	No	Negative at birth and day 2 of life	Alive and healthy. Doing well at 6 months age
										2.2 kg	None		

Case 2

**Figure 2:**
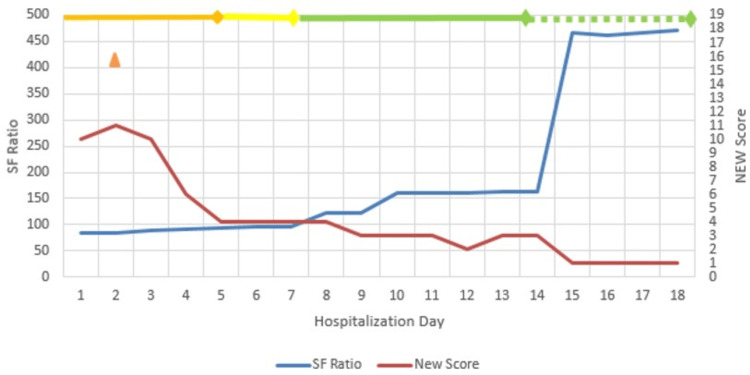
Clinical timeline of Case 2. SF ratio = SpO_2_/FiO_2_; NEW Score: National Early Warning Score

A 28-year-old G2P1L1 presented at 23 weeks gestation with fever and acute shortness of breath of two days' duration. RTPCR was positive for SARS-CoV-2. She was agitated and confused with SpO_2_ of 85% on room air at admission. With a National Early Warning Score (NEW Score) of 10, she was admitted in ICU. Oxygen support was administered through NIV (Figure 2). Intravenous remdesivir was started the next day after proper counseling and informed consent. Intravenous steroids, LMWH, and broad-spectrum antibiotics were also initiated as a part of standard care. Her SpO_2_ and leucocytosis showed dramatic improvement. By day 5, SpO_2_ ranged above 95%, and she was shifted to HFNC on day 6 and NRM on day 8. 

She developed elevation of hepatic transaminases on day 5 of hospital stay (Table 2). Because the elevation was mild, the last dose of remdesivir was continued and ursodeoxycholic acid (UDCA) was started, which led to recovery within 48 hours. No other adverse effects following remdesivir use were observed. LMWH was discontinued on day 14, and she was discharged in a satisfactory condition on day 18. Follow-up antenatal ultrasound did not reveal any structural malformations in the fetus. The patient delivered a female baby via cesarean section at 37 weeks two days gestation for oligohydramnios and asymmetrical fetal growth restriction at a private nursing home. The baby's birth weight was 2.3 kg. The Apgar score was 9, 10. The baby is currently 24 months old and doing well. 

**Table 2:** Laboratory investigations. Hb: hemoglobin; TLC: total leucocyte count; N: neutrophils; L: lymphocytes; BU: blood urea; SC: serum creatinine; AST: aspartate aminotransferase; ALT: alanine transaminase; INR: international normalized ratio; RBS: random blood sugar; CRP: C-reactive protein; LDH: lactate dehydrogenase

Case no.	Days	Hb (g/dl)	TLC (/mm^3^)	Platelets (lacs/ mm^3^)	N (%)	L (%)	BU (mg/dl)	SC (mg/dl)	Bilirubin (mg/dl)	AST/ALT (U/L)	INR	RBS (mg/dl)	CRP (mg/dl)	D-dimer (ng/ml)	Procal (ng/ml)	LDH (U/L)	Ferritin (ng/ml)
Case	D1	11.0	14,220	2.07	94.2	2.2	9.2	0.7	0.4	30/14	1.02	70	3.6	14,200	0.16	2465	47.9
1	D3	10.2	12,330	2.27	85.7	7.7	5.3	0.6	0.5	34/10	1.01	95	-	-	0.24	-	-
	D5	10.9	13,660	2.16	83.2	9.0	6.0	0.5	0.4	42/26	1.00	96	3.4	7250	-	2070	-
	D8	11.6	12,290	2.76	83.9	9.3	16	0.2	0.5	40/30	1.02	120	-	-	0.08	-	37.2
	D15	12.2	15,520	2.00	86.2	8.3	27	0.4	0.5	46/70	1.01	106	-	4200	-	940	32.5
	D29	12.9	15,150	3.37	77.0	15.2	37	0.4	0.4	40/140	1.2	133	1.6	1740	0.02	526	33.9
Case	D1	12.6	14,200	1.26	93.8	2.3	33	0.3	0.4	67/76	1.2	121	2.4	7620	0.36	-	26.0
2	D3	13.1	13,650	1.40	94.2	1.8	28	0.5	0.5	86/102	1.07	108	-	-	-	-	-
	D5	13.4	13,100	1.36	85.6	10.7	30	0.6	0.5	126/152	1.1	116	-	3450	0.09	1470	-
	D8	12.9	7570	1.42	82.2	13.8	22	0.2	0.4	54/60	1.1	132	1.0	-	-		
	D15	13.0	9800	1.25	78.2	17.7	24	0.4	0.4	42/56	1.1	117	-	970	-		
Case	D1	8.8	8900	2.12	92	4.2	14	0.82	0.71	80/44	1.20	88	2.6	1070	0.09	-	-
3	D3	9.1	12,700	2.06	93	3.9	15	0.9	0.6	70/52	0.99	105	-	-	-	-	-
	D5	9.0	13,290	2.20	90	3.8	18	0.7	0.8	66/56	0.98	96	2.4	1560	0.4	-	-
Case	D1	10.4	9550	2.20	89.7	8.1	108	0.7	0.77	58/106	0.98	116	2.4	1470	0.09	1343	41.0
4	D3	11.4	37,010	1.20	94.2	2.2	114	1.6	1.02	76/97	1.16	120	2.6	-	-	-	-
	D5	11.6	21,170	1.47	93.7	5.4	126	2.3	1.28	137/113	1.28	82	2.4	2260	-	-	-

Case 3

**Figure 3:**
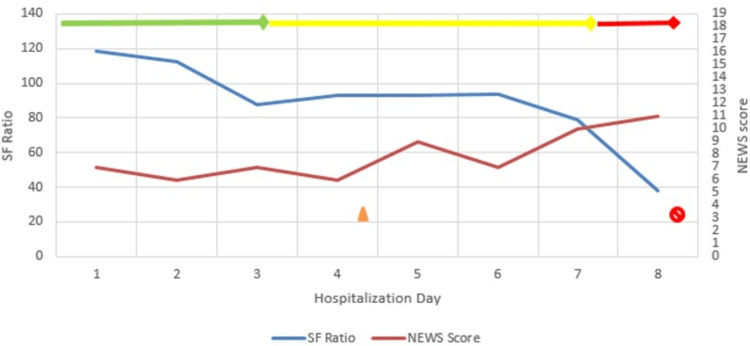
Clinical timeline of Case 3. SF ratio = SpO_2_/FiO_2_; NEW Score: National Early Warning Score

A 35-year-old G3P2L2 with previous two caesarean deliveries presented at 29 2/7 weeks with acute onset dyspnea for three days. She had tested positive on RTPCR for SARS-CoV-2 three days ago and was on ward-based care in another facility. Admission SpO_2_ was 92% on room air (Figure 3). 

Obstetric exam was unremarkable. Supplemental low-flow oxygen and standard supportive care was started (Table 1). SpO_2_ subsequently remained above 93%, and the respiratory rate ranged between 20 and 24/min. On day 3, SpO_2_ levels dipped to 70% on NRM. She had persistent tachycardia and was stepped up to ICU care. Intravenous steroids, remdesivir, broad-spectrum antibiotics, LMWH, and prone positioning were initiated, following which oxygen saturation stabilized above 90%. However, on day 6, she became increasingly tachypneic and hypertensive. SpO_2_ levels fell to 64-70% and she was intubated for invasive mechanical ventilation (IMV). The patient developed refractory hypoxemia, did not respond to maximum ventilatory support, and expired on day 7 following a cardiopulmonary arrest (Table 1). 

Case 4

A 27-year-old G6P2L2A3 SARS-CoV-2 RTPCR-positive woman presented at 34 5/7 weeks with fever and tachypnea for one day (Table 1). SpO_2_ on room air was 70%. Due to non-availability of ICU and high-dependency unit (HDU) beds, the patient was managed in a dedicated COVID-19 ward. She was put on NRM and supportive care, following which she showed symptomatic improvement with SpO_2_ ranging above 90%. Obstetric examination was unremarkable. On day 2, SpO_2_ declined to 80% and she was escalated to ICU care with HFNC. Intravenous steroids, broad-spectrum antibiotics, remdesivir, and LMWH were initiated, and the patient was clinically stable for two days with SPO_2_ ranging above 90%. On day 4, the patient went into spontaneous preterm labor and had an uneventful vaginal delivery. In the early post-partum period, SpO_2_ fell to 75%, and due to increasing need for respiratory support, the patient was hiked to NIV. Transthoracic echocardiography was negative for cardiomyopathy or pulmonary embolism. In view of progressively increasing leukocytosis and acute kidney injury, antibiotics were stepped up after a nephrology consult (Table 2). On day 5, she had an episode of epistaxis. The coagulation profile was normal, and she responded to conservative management.

Due to the inability to maintain oxygen saturation and worsening acidosis, she was intubated and IMV was initiated on day 5. There was a mild elevation in hepatic transaminases on day 5, likely due to septicemia-induced multi-organ dysfunction (Table 2). Despite maximum ventilatory support, she progressively deteriorated and succumbed to a cardiopulmonary arrest on day 6. Her healthy born male neonate tested negative for RTPCR and was discharged to routine care and follow up. The baby is doing well at the 18-month follow-up.

## Discussion

Remdesivir, a ribonucleic acid (RNA) nucleoside analogue polymerase inhibitor, has been investigated as a potential therapeutic option due to its broad inhibitory activity against viruses of the families *Coronaviridae* (including SARS-CoV, MERS-CoV, and certain bat coronaviruses), *Paramyxoviridae*, and *Filoviridae* [7-10]. It has demonstrated in vitro activity against SARS-CoV-2 [11]. In a rhesus macaque model of SARS-CoV-2 infection, when remdesivir was initiated soon after inoculation, the remdesivir-treated animals had a lower virus load in the lungs and lesser pulmonary insult than controls [12]. Its active metabolite GS-441524 displays linear pharmacokinetics and a prolonged intracellular half-life of greater than 35 hours. It accumulates in the peripheral blood mononuclear cells, suggesting that a loading dose would accelerate the achievement of a steady state [8] Therefore, a dosing regimen of 200 mg intravenous loading dose followed by 100 mg intravenous once daily for five to 10 days is used clinically [13]. It is metabolized in the liver and undergoes renal and biliary excretion. There is an antagonistic effect of hydroxychloroquine sulphate and chloroquine phosphate on intracellular activation and antiviral activity of remdesivir, and therefore co-administration is not advised [10]. 

The most definitive evidence for remdesivir use in COVID-19 comes from the Adaptive COVID-19 Treatment Trial 1 (ACTT-1), which has shown a shorter recovery time (10 days versus 15 days), lesser mortality at day 15 (6.7% versus 11.9%) and day 29 (11.4% versus 15.2%), and lesser adverse events (24.6% versus 31.6%) with intravenous remdesivir treatment as compared to placebo. Pregnant and lactating women were, however, excluded from the study [14]. Meanwhile, the WHO Solidarity trial, an open-label study, concluded that remdesivir did not decrease in-hospital mortality compared to the "standard of care" [15]. Other studies have observed mixed results with intravenous remdesivir use, but most are handicapped by methodological limitations. The only large multicentric study describing the outcomes of compassionate use of remdesivir in 67 pregnant and 19 puerperal women has demonstrated higher recovery rates and lower rates of severe adverse events with remdesivir use [16]. The median interval between the onset of symptoms and initiation of remdesivir was nine days. At the 28-day follow-up, oxygen requirement was reduced in 96% pregnant and 89% postnatal women. Remdesivir was well tolerated. There was one maternal death and no neonatal death. Another study describing remdesivir use in 17 pregnant women with moderate to severe COVID-19 pneumonia has noted that delaying remdesivir for more than 48 hours after admission (heart rate (HR) 2.32, 95% confidence interval (CI) 1.45-4.16) and more than four days' duration of symptoms prior to hospitalization (HR 1.65, 95% CI 1.27-3.50) had an inverse association with clinical recovery. Elevated transaminases were seen in 33% (8/24) women [17]. Evidence has also been accumulated from multiple isolated case reports and series describing remdesivir use [18]. Most have generally observed a favorable response with a faster recovery in moderate to severe COVID-19 pneumonia in pregnancy. 

Animal studies have failed to demonstrate any adverse embryo or fetal defects in pregnant rats and rabbits at non-toxic dosing levels. Remdesivir and its metabolites were detected in the plasma of nursing pups at levels of 1% maternal exposure on lactation day 10. There is no available data on the excretion in breast milk, effects on breast-fed human infants, or effects on milk production [19]. The only randomized controlled trial investigating remdesivir as one among multiple treatment therapies for Ebola virus disease recruited six pregnant women to the remdesivir arm. There is no mention on any drug-related maternal or fetal adverse effects. The remdesivir arm was, however, prematurely terminated due to a lack of sufficient efficacy [20]. Remdesivir is not considered a potential mutagenic or clastogenic agent as per in vitro and in vivo studies performed to assess its genotoxic potential [21].

Remdesivir had been granted EUA for the treatment of suspected or laboratory-confirmed COVID-19 in adults and children who are hospitalized with severe disease, requiring supplemental oxygen, or requiring mechanical ventilation or extracorporeal membrane oxygenation (ECMO) in May 2020. The issuance allowed the use of the same dosing regimen in pregnant and parturient women as in the general adult population [22]. In October 2020, approval was given for the use of remdesivir in adult and pediatric COVID-19 patients requiring hospitalization [23]. However, despite being an extremely vulnerable subset, pregnant women have traditionally, and also specifically with respect to SARS-CoV-2, been excluded from clinical trials. Of the COVID-19 trials in 10 international clinical registries, 75% have excluded pregnant women specifically [24].

As of February 2021, the US National Institute of Health has funded a phase one, non-randomized, open-label opportunistic pharmacokinetic and safety study in pregnant and non-pregnant women of child-bearing age group who are on remdesivir therapy [25]. In all cases, in keeping with the national guidelines, remdesivir was initiated within seven days of onset of symptoms. Of the four patients treated, there were two mortalities. Two patients survived and were subsequently discharged in satisfactory condition. In case 1, in addition to standard care, tocilizumab was administered to stem the cytokine storm. This patient showed steep clinical improvement on initiation of this therapy. Of the four patients, two women received remdesivir in the antenatal period, while two received it in the postnatal period. We followed all three live born infants of the COVID-19-positive mothers. Two of these infants were exposed to remdesivir in-utero. A detailed physical and developmental evaluation of these infants has revealed no obvious short-term adverse effects of remdesivir exposure in-utero. At the six-month follow-up, both infants who were exposed to remdesivir in-utero were doing well. In our experience, remdesivir use seems to be safe for fetuses and infants. It is most effective when started early in the viremic phase.

## Conclusions

Early initiation of remdesivir in pregnancy in the viremic phase seems to provide some advantage in the survival outcome. In pregnant women with raised inflammatory markers (C-reactive protein (CRP) and IL-6), remdesivir use along with tocilizumab may improve survival outcome. Its use may be associated with transient elevation in hepatic transaminases in some cases. No detrimental effects on the ongoing pregnancies, fetuses, or neonates have been observed. Further large-scale studies may provide more conclusive evidence.

## Figures and Tables

**Figure 4:**
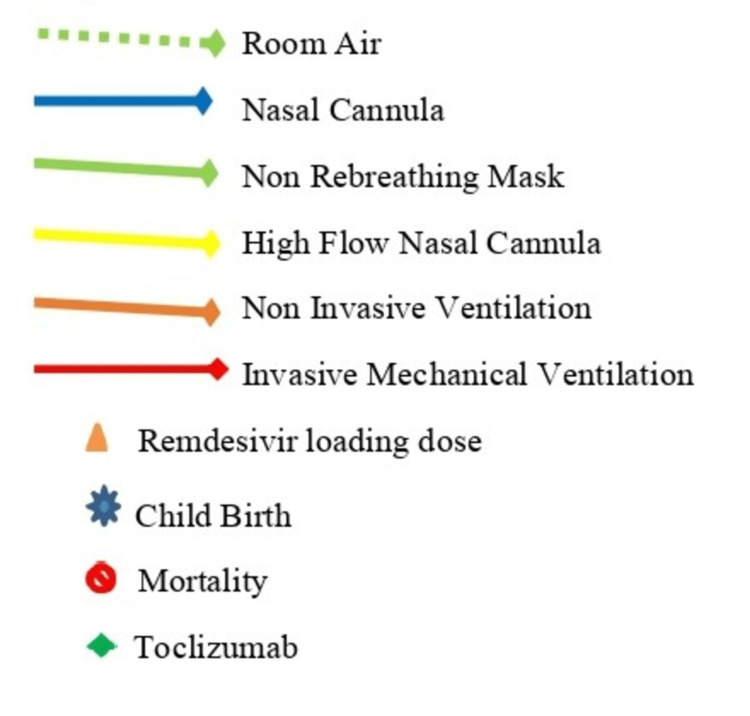
Key for Clinical Intervention and Outcome.
